# Systematic Review of Clinical Outcome Parameters of Conservative Treatment of Adolescent Idiopathic Scoliosis Patients

**DOI:** 10.3390/jcm14041063

**Published:** 2025-02-07

**Authors:** Tuğba Kuru Çolak, Betül Beyza Durmuş, Ece Zeynep Saatçı, Engin Çağlar, Burçin Akçay, Santiago Lasa Maeso

**Affiliations:** 1 Department of Physiotherapy and Rehabilitation, Faculty of Health Sciences, Marmara University, 34854 Istanbul, Turkey; beyzadurmus@marun.edu.tr (B.B.D.); ecesaatci@marun.edu.tr (E.Z.S.); 2 Department of Physiotherapy and Rehabilitation, Faculty of Health Sciences, Fenerbahce University, 34854 Istanbul, Turkey; engin.caglar@fbu.edu.tr; 3 Department of Physiotherapy and Rehabilitation, Faculty of Health Sciences, Bandırma Onyedi Eylül University, 10200 Balıkesir, Turkey; bakcay@bandirma.edu.tr; 4 Clinica De La Escoliosis, Montevideo 11600, Uruguay; sbp.latam@gmail.com

**Keywords:** brace, exercise, outcome measurements, scoliosis, physiotherapy, rehabilitation

## Abstract

**Background:** The purpose of this systematic review was to investigate the clinical outcome measures, either in separation or in conjunction with the Cobb angle, that were employed in randomized controlled trials to evaluate posture and spinal deformity. **Methods:** The PubMed, PEDro, OVID, and Scopus electronic databases were used to search for published articles from January 2004 to May 2024. All the searches included English language studies. Keywords were determined as “scoliosis, exercise, physical activity, brace, bracing, conservative treatment, rehabilitation, physiotherapy and physical therapy”. **Results:** The most commonly used clinical evaluation parameters other than the Cobb angle in the studies were the ATR and surface topography measurements. The most commonly used assessment methods were POTSI asymmetry assessment performed with measurements made on photographs, the WRVAS, a deformity perception assessment evaluated with different photographs, and the SRS-22 scale, a disease-specific quality of life assessment. **Conclusions:** New developments in the treatment of scoliosis have brought about alternative measurement techniques that offer a more comprehensive view of patient outcomes. As a result of the review of the literature, it has emerged that radiation-free, valid, and reliable multidimensional evaluations should be performed in follow-up and treatment efficacy evaluations after initial diagnosis in adolescent idiopathic scoliosis.

## 1. Introduction

Scoliosis is defined as a three-dimensional deformity of the spine. Clinically, the most common type of scoliosis is adolescent idiopathic scoliosis (AIS). Although scoliosis is a three-dimensional deformity, the diagnostic criterion is a Cobb angle of more than 10 degrees, which is evaluated in two dimensions in the frontal plane on standing AP orthorontographs [1,2].

Although the Cobb angle was defined in 1948, it is still used as the gold standard evaluation method in diagnosing scoliosis, determining treatment options and effects, and evaluating progression [3,4]. While the Cobb angle is a widely used parameter to assess the severity of scoliosis, its direct impact on patients’ quality of life remains a topic of discussion. Although the Cobb angle is frequently used, there is insufficient data to conclude that better radiological results are associated with long-term improvements in scoliosis patients’ function, quality of life, self-image, and pain [5]. According to published reports, adults with AIS who have not received treatment are highly functional and productive at a 20th, 28th, and 50th-year follow-up even though they have a large Cobb angle. Back pain and cosmetic concerns are the only physical impairments caused by untreated AIS [6,7]. Additionally, the overall quality of life and the Cobb angle have a weak relationship [8].

According to SOSORT and SRS criteria, a decrease of more than 5 degrees in the Cobb angle is considered an improvement, and an increase of more than 5 degrees is considered progression [9,10]. Although Cobb angle measurements are still very important for professionals working in the specialty of scoliosis, it should be kept in mind that there may be differences even between the Cobb measurements of the same person. It has been reported that there can be a difference of 3.5–7.1 degrees between two measurements when measured manually and 3.2–6.1 degrees when measured using computer systems [11]. A measurement error of 5 degrees is very important as it will change the patient’s treatment program [10].

Another important point is the radiation exposure of patients due to X-rays taken to evaluate the Cobb angle. Up to 40 to 50 X-rays may be required for certain patients, particularly those who are diagnosed at a younger age and are receiving early and continuous treatment [12]. After evaluating 13 females who had 22 X-rays over a 3-year period, Nash et al. calculated an increase in breast cancer of 140 to 290 per million and an increase in organ cancer of 3.4 to 15 per million [13] Levy et al. assessed 2039 patients referred to a large pediatric hospital between 1925 and 1965 who received an average of 12 and 10 X-rays, respectively, and calculated an excess cancer risk of 42 to 238 cases per 100,000 for women and 14 to 79 cases per 100,000 for men [14].

While the majority of scoliosis cases (e.g., adolescent idiopathic scoliosis, congenital scoliosis with single formation defects) may not result in significant health-related impairments in many individuals, even if untreated, the potential for psychosocial and functional consequences should not be overlooked. In this context, the trunk deformity caused by scoliosis often becomes a central concern for patients [15,16,17]. It is therefore advisable to focus more on the cosmetically significant changes in the shape of the trunk as an outcome parameter, as these can lead to impairments in quality of life under certain circumstances.

Considering all these factors, other outcome measurement methods to assess deformity, spine, and posture are gaining importance. The clinical evaluation of individuals with scoliosis involves a comprehensive assessment that includes anamnesis, physical examination, inspection, the forward bending test, general anthropometric measurements, maturation assessment, the evaluation of leg length discrepancy, the assessment of the plumb line (midline alignment), trunk rotation angle measurement, gibbosity evaluation, lumbar asymmetry assessment, the assessment of spinal rigidity and flexibility, as well as endurance and muscle strength testing. Furthermore, it incorporates subjective assessment methods such as aesthetic evaluation, posture analysis, stress scales, and quality of life questionnaires [18].

The forward bending test has been reported to have a sensitivity of 92% to 100% for detecting curvatures exceeding 20 degrees and a specificity of 60% to 91% [18]. Among these evaluation methods, height measurement, as part of general anthropometric assessments, is recommended with 95.6% agreement by SOSORT professionals [19]. As part of the maturation assessment, the evaluation of menarche status is suggested by members of SOSORT and the Schroth Best Practice Academy [18,19,20]. The SOSORT consensus report recommends assessing the C7 plumb line with 73.9% agreement [19]. The evaluation of the angle of trunk rotation (ATR) using a scoliometer has been reported to have excellent intra-rater reliability and very good inter-rater reliability. A minimum of a 5 degree ATR detected by the scoliometer is considered a good criterion for identifying a Cobb angle of 20 degrees or more [21]. While studies on surface topography systems have reported positive results related to the Cobb angle, some publications have highlighted issues such as false positive results, poor sensitivity, and reliability. Aesthetic appearance and the perception of postural appearance are among the most critical parameters to be addressed in the treatment of adolescent idiopathic scoliosis. Various tools have been developed to assess aesthetic and deformity perception, including the Walter Reed Visual Assessment Scale (WRVAS), the Spinal Appearance Questionnaire (SAQ), and indices such as the Anterior Trunk Symmetry Index (ATSI) and the Posterior Trunk Symmetry Index (POTSI) [18].

The aim of this systematic review was to identify and highlight the most commonly used and preferred clinical outcome measures used in randomized controlled studies to assess spinal deformity and posture, either alone or in combination with the Cobb angle.

## 2. Materials and Methods

### 2.1. Search Strategy

A search was carried out in the databases PubMed, PEDro, OVID, and Scopus provided by the remote access system of the Marmara University Library and Documentation Department. The search strategy was constrained to the period of January 2004 to May 2024. Only articles written in English were included. The search strategy used was as follows: (scoliosis [MeSH Terms] OR scoliosis [Title/Abstract]) AND (exercise [MeSH Terms] OR exercise [Title/Abstract] OR “physical activity” [Title/Abstract] OR brace [Title/Abstract] OR bracing [Title/Abstract] OR “conservative treatment” [Title/Abstract] OR rehabilitation [Title/Abstract] OR physiotherapy [Title/Abstract] OR “physical therapy” [Title/Abstract] OR treatment [Title/Abstract]) AND Randomized Controlled Trial [pt]. We employed comparable search terms or synonymous terms in alternative databases.

This systematic review was conducted in accordance with the Preferred Reporting Items in Systematic Reviews and Meta-Analyses (PRISMA) Statement.

### 2.2. Study Selection and Criteria

The studies were chosen according to predetermined criteria for inclusion and exclusion after searching through the databases. Initially, the identification of studies relied on screening titles and abstracts, which was followed by full-text evaluation and the eventual inclusion of studies. Three reviewers conducted the screening process for the articles. Criteria for inclusion and exclusion were based on the PICOS format (Table 1) [22]. For the population criterion, individuals who were diagnosed with adolescent idiopathic scoliosis between the ages of 9 and 19 and did not have any other concomitant disease were included, regardless of gender. Studies including radiologically diagnosed cases were enrolled in this systematic review. The interventions included were determined as deformity corrective interventions for the spine (exercise, brace, physiotherapy, and other interventions). Studies performed before or after surgical intervention were excluded. Studies including surgical and post-surgical interventions were excluded. The outcome of our interest included any measurement method that evaluates scoliosis, spine, deformity, or posture other than the Cobb angle. Only randomized controlled trials (RCTs) were examined for this study. A limitation was placed by including only English language studies published from January 2004 to May 2024.

### 2.3. Data Extraction and Data Synthesis

After reviewing all of the final included studies, data extraction was performed in Microsoft Excel independently by three authors. A reliability assessment of the studies was made using the PEDro scale [23]. After data extraction, narrative synthesis was conducted. Heterogeneity tests and meta-analysis of the studies were not performed.

## 3. Result

### 3.1. Study Selection

**Figure 1:**
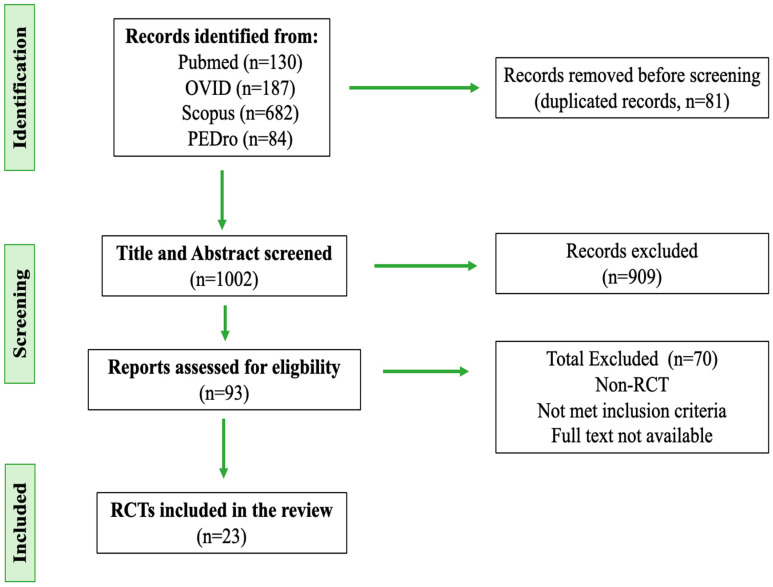
Study selection, flow diagram.

A total of 1083 articles were retrieved from the four databases. A total of 81 articles were excluded due to duplication. A total of 909 articles were excluded after reading the title and abstract. Finally, 23 articles were included in the study after reviewing the remaining 93 articles (Figure 1) (Table 2).

### 3.2. Study Characteristics

In all included studies, it was ensured that the diagnosis of scoliosis was made by radiographic evaluation. All articles were in English. The oldest study included in the study was from 2005 [24], and the most recent study was from 2024 [45]. Seven of the included studies were conducted in Turkey, four in China, and three in the USA.

A total of 1252 participants were assessed in the studies. Of the participants, 1086 (86.74%) were female. Seven of the studies [24,26,31,35,38,40,41] included only females. In one of the studies that included [34], the participants’ gender was not clearly defined.

The most commonly evaluated outcome, other than the Cobb angle, was the trunk rotation angle (ATR). In 15 studies [26,27,29,30,32,33,36,37,38,40,42,43,44,45], the ATR was evaluated as the primary or secondary outcome measurement. The apical vertebral rotation (AVR) was evaluated on X-ray in one study [24]. Sagittal balance was evaluated in three studies [6,26,42], and coronal balance in two studies [6,42].

Surface topography was used as a non-invasive imaging technique to evaluate deformity in four studies [25,26,27,28]. In one study, a computer system was used to assess posture [35], and in another study, a posture assessment application [40] was used.

The Posterior Trunk Asymmetry Index (POTSI) was used to evaluate trunk asymmetry in four studies [30,31,33,40]. The Anterior Trunk Asymmetry Index (ATSI) was used in one study [40].

In addition to objective measurements, the studies also used some scale assessments as outcome measures. The Walter Reed Visual Assessment Scale (WRVAS) was the most commonly used visual assessment scale and was used in four articles [31,33,37,40]. Two studies used the Scoliosis Appearance Questionnaire (SAQ) [34,36] and the other two used the Trunk Appearance Perception Scale (TAPS) [30,41].

Improved quality of life was evaluated as an outcome in 11 studies. The most used scale to assess quality of life was The Scoliosis Research Society-22r (SRS-22r). In 10 studies [27,30,31,33,34,36,37,41,42,43], SRS-22r was used, and in one study, SRS-23 [29] was used to assess quality of life.

Quality of life was assessed in one study [6] by using the Pediatric Quality of Life Inventory (PedsQL), and in one study using the EuroQol 5-Dimensions Youth (EQ-5D-Y) [33].

### 3.3. Methodological Quality of Included Studies

The PEDro scale was ranked from 3 to 8, with an average score of 6.21/10 for overall articles. Five of the studies [6,24,25,32,34] scored four or less on the PEDro scale (Table 3). These scores indicate low methodological quality. Seventeen studies scored between 5 and 7, indicating moderate methodological quality. Only three studies had high methodological quality. These three studies [37,38,42] scored eight and above (Table 4). The most important reason for the low scores of the studies was the lack of blinding of the physiotherapists, patients, and evaluators included in the study.

### 3.4. Interventions Used in the Studies

In the reviewed articles, various interventions were employed to manage AIS. The most commonly used intervention across the studies was Schroth exercises, which demonstrated significant positive outcomes in improving both the ATR and quality of life [27,43,44]. Schroth exercises were particularly effective, with multiple studies [27,29] highlighting improvements in spinal deformities and patient functionality. Additionally, bracing was another widely used intervention, with various forms such as the Boston brace, the TLSO brace, and innovative braces designed using CAD/CAM technology [24,28]. For instance, Wong et al. found that CAD/CAM braces were more effective than manually produced braces [24].

Several studies also combined Schroth exercises with other interventions such as balance training and hippotherapy. Abdel-Aziem et al. showed that combining hippotherapy with Schroth exercises improved balance and postural asymmetries [39]. Furthermore, recent technological advancements have introduced the use of 3D-printed orthoses. Lin et al. reported significant improvements in initial flexibility and quality of life with these orthoses [41].

Control groups in the studies varied but commonly included standard treatments or observational groups to measure the effectiveness of the interventions. Many control groups were provided with standard braces, such as the traditional Boston brace, against which newer or more specialized interventions were compared [28]. In some studies, the control groups engaged in conventional exercise programs in contrast with more specialized therapeutic exercises [31]. Several studies also included control groups that received no active treatment to measure the natural progression of scoliosis without intervention [6].

Overall, the Schroth exercise program was the most frequently used intervention due to its proven efficacy in improving both physical deformity and quality of life in AIS patients. For the control groups, standard bracing and conventional exercise programs were most commonly preferred to provide a baseline for comparing the effectiveness of newer or more intensive treatment methods.

## 4. Discussion

This systematic review aimed to identify and present the most commonly used clinical assessment methods, apart from Cobb angle measurements, in randomized controlled trials (RCTs) evaluating the results of conservative treatments in patients with adolescent idiopathic scoliosis. According to the results of our study, ATR assessment was the most used non-invasive and objective deformity assessment method. Surface topography was the most widely used technological system to assess posture and deformity. The POTSI, in which asymmetry is evaluated with certain calculations on the photograph, was the other most commonly used asymmetry assessment. The WRVAS was the most preferred visual asymmetry assessment scale. The SRS-22 instrument was the most commonly used quality-of-life assessment questionnaire.

### 4.1. Cobb Angle and Clinical Significance

The Cobb angle remains the gold standard for assessing the severity of scoliosis. However, its clinical significance extends beyond simply quantifying spinal curvature. While the Cobb angle is crucial for diagnosing scoliosis and determining treatment strategies, its impact on long-term functional outcomes, quality of life, and self-image is less clear. Several studies indicate that untreated AIS patients with significant spinal deformities can maintain high levels of functionality and productivity [6]. This finding suggests that improvements in the Cobb angle do not necessarily correlate with better patient-centered outcomes, such as quality of life and physical functioning [8].

The frequent use of X-rays for Cobb angle assessment raises concerns about radiation exposure, particularly in young patients. Studies have shown that repeated radiographic examinations can increase the risk of radiation-induced conditions, including cancer [12]. Non-invasive assessment methods and computer-assisted measurement tools are being created and should be used more frequently to reduce these risks.

As the authors of the study, we would like to emphasize the importance of the Cobb angle and radiological evaluation in the differential diagnosis of idiopathic scoliosis. A postural asymmetry or an ATR value above the cut-point in an evaluation with a scoliometer cannot confirm the diagnosis of scoliosis. The patient may have a congenital deformity or a hemidystrophic thorax [1,46]. However, it is clear that alternative methods are needed for follow-up and evaluation after diagnosis.

### 4.2. Outcome Measurement Methods

Nowadays, measurement methods that provide a more holistic view of patient outcomes are preferred in scoliosis management. Many patients and their families care more about the cosmetic appearance than the angle measured on the X-ray. One of the main objectives of scoliosis treatment has been identified as improving one’s aesthetic appearance, and quality of life is also significantly impacted by one’s perception of one’s body [47,48].

ATR measurement has been shown to be highly correlated with the Cobb angle. It is a simple, cost-effective, safe, valid, and reliable method for clinical use. A change in the ATR is associated with a change in the Cobb angle and the asymmetries that the patient recognizes in the body [49,50,51,52]. Therefore, it can be suggested that ATR measurements made with a scoliometer can be used in patient evaluation and follow-up.

When the randomized controlled studies were examined, it was determined that parameters such as the trunk rotation angle (ATR), the posterior trunk asymmetry index (POTSI), and surface topography assessment gained importance. For example, the ATR, in addition to the two-dimensional Cobb angle measurement, provides an assessment of the change of spinal deformity in the horizontal plane.

Surface topography techniques were identified as a preferred assessment method in the studies. This method has been reported to be valid and reliable, especially in evaluating sagittal and frontal plane asymmetries [53]. Surface tomography and ATR measurements may have great potential in the evaluation of scoliosis, especially in reducing radiation exposure and in cosmetic evaluations. Research indicates that integrating these measures could yield a more precise and all-encompassing evaluation of scoliosis for patient assessment and treatment effectiveness.

### 4.3. Cosmetic Appearance and Quality of Life

The psychosocial impact of scoliosis, particularly concerning cosmetic appearance and quality of life, is significant. The evaluation of cosmetic appearance and quality of life is suggested as a parameter that should be included in the management of scoliosis [10]. Trunk deformity is an important symptom of idiopathic scoliosis and has also been reported to present a major problem for patients [10]. In the literature, various scales have been developed to evaluate patients’ and families’ perceptions of deformity and physical appearance. In the studies included in this systematic review, it was determined that the Walter Reed Visual Assessment Scale (WRVAS) scale, which evaluates deformity with seven different photographs, was used the most. In addition, it was determined that the TAPS, which includes deformity assessment with photographs, and SAQ scales, which include both photographs and questions, were also preferred in the studies. These tools are crucial as they address the subjective aspects of scoliosis, often overlooked in clinical practice.

Quality of life assessment is also an important parameter in the treatment and follow-up of scoliosis because the treatment of scoliosis can be quite long-term, especially in individuals diagnosed at a young age [10]. The most preferred quality of life assessment in the studies was the SRS-22 questionnaire, which includes 22 questions and five subgroup assessments.

### 4.4. Treatment Interventions and Outcomes

Nearly all studies included in this review focused on conservative treatment methods, excluding surgical interventions. The interventions evaluated include Schroth exercises, 3D-printed orthoses, balance training, and hippotherapy. Schroth exercises demonstrated significant positive outcomes across multiple studies. These exercises have been shown to significantly improve the Cobb angle, the ATR, and quality of life [27,29,44]. Other interventions, such as balance training combined with Schroth therapy, also showed promising results in improving spinal deformities and patient functionality [43].

Specifically, Abdel-Aziem et al. [39] found that a combination of hippotherapy and Schroth exercises improved balance and postural asymmetries. Wong et al. [24] demonstrated that the CAD/CAM method for spinal orthosis was more effective than the manual method in managing AIS. Lin et al. [41] reported significant improvements in initial flexibility and quality of life with the use of 3D-printed orthoses, as measured by SRS-22r and TAPS.

### 4.5. Methodological Quality and Future Research Directions

The methodological quality of the studies included in this review, as assessed by the PEDro scale, ranged from low to moderate. Common methodological weaknesses included a lack of blinding and allocation concealment. However, it is important to note that all the studies included in this systematic review were randomized controlled trials, which are positioned at the top of the evidence hierarchy. Since our focus was not on treatment effectiveness but rather on analyzing the assessment measures used, the low-quality scores of some articles were considered less critical for the purposes of this review. Future research should focus on high-quality RCTs that incorporate both objective and subjective outcome measures, providing a more comprehensive understanding of the impacts of scoliosis treatment [23].

### 4.6. Limitations

This systematic review has several limitations. The heterogeneity in the measurement methods and outcome assessment criteria used in the studies may reduce data homogeneity. Additionally, the focus on English-language publications may introduce language bias and result in the omission of relevant studies published in other languages.

## 5. Conclusions

Our findings underscore the diversity and effectiveness of various measurement methods and interventions employed in scoliosis management, highlighting the need for a multifaceted approach to patient assessment and treatment. The most widely used non-invasive objective deformity assessment technique, according to the findings of our study, was ATR evaluation. In order to evaluate posture and deformity, surface topography was the most commonly utilized technical system. The POTSI was the other most widely used asymmetry assessment method. In this method, asymmetry is assessed using specific calculations on a picture. The visual asymmetry assessment scale that was most favored was the WRVAS. Questionnaires for quality-of-life assessments were most frequently administered using the SRS-22 scale.

## Figures and Tables

**Table 1:** The inclusion and exclusion criteria of the study.

	Inclusion	Exclusion
Population	Female/Male 9–19 years Radiological diagnosis of AIS	Having any other concomitant disease
Intervention	Physiotherapy Exercise Brace treatment Other conservative therapies	Surgical operation
Comparator	Any comparator	Surgery Pre/post-operation interventions
Outcome	Any measurement method that evaluating scoliosis, spine, or deformity	Not include a spine-related measurement
Study	RCT	Non-randomized study Qualitative study Review Analytical study Protocol
Language	English	Non-English
Year	2004–Present	Before 2004

**Table 2:** Study characteristics (studies are listed by year).

Study	Country	Gender n (M/F)	Age	Risser Sign	Cobb Angle Assessment	Outcome Measurements	Control Group	Intervention Group
Wong et al., 2005 [24]	China	40 (0/40)	10–14	Risser Sign < 2	Yes	AVR	Brace produced by manual method	Brace produced by the CAD/CAM method
Labelle et al., 2007 [25]	Canada	48 (2/46)	10–16	Risser Sign ≤ 3	Yes	Thoracic kyphosis Lumbar lordosis Surface topography	Boston brace	Boston brace designed and adjusted using a computer-aided 3-dimensional tool
Hasler et al., 2010 [26]	Switzerland	20 (0/20)	15.2–18.5	-	Yes	ATR Surface topography Spine flexibility Plumb line Sagittal balance Thoracic kyphosis Lumbar lordosis	Observation	Osteopathy
Weinstein et al., 2013 [6]	USA	242 (21/221)	10–15	Risser Sign 0–2	Yes	Coronal balance Sagittal balance Thoracic kyphosis Lumbar lordosis PedsQL	Observation	TLSO Brace
Monticone et al., 2014 [27]	Italy	110 (30/80)	>10	Risser Sign < 2	Yes	ATR SRS-22r	General exercises aimed at spinal mobilization	Active self-correction, task-oriented exercises
Cobetto et al., 2016 [28]	Canada	40 (5/35)	10–16	Risser Sign 0–2	Yes	Thoracic kyphosis Lumbar lordosis Surface topography	Conventional TLSO Brace	New TLSO brace (NewBrace)
Kuru et al., 2016 [29]	Turkey	45 (6/39)	10–18	Risser Sign 0–3	Yes	ATR Maximum height of the hump Waist asymmetry SRS-23	No treatment	I1: Schroth exercises under physiotherapist supervision I2: Schroth home exercise program
Gur et al., 2017 [30]	Turkey	25 (1/24)	10–16	Risser Sign 0–5	Yes	ATR POTSI TAPS SRS-22	Traditional rehabilitation	Core stabilization in addition to traditional rehabilitation
Yagci et al., 2018 [31]	Turkey	20 (0/20)	10–16	Risser Sign 1–3	Yes	ATR POTSI WRVAS SRS-22	Conventional exercise	Basic body awareness therapy
Sarkisova et al., 2019 [32]	USA	64 (11/53)	10–17	-	Yes	ATR	Front plank	Side plank
Yagci et al., 2019 [33]	Turkey	30 (0/30)	>12	Risser Sign 2–3	Yes	ATR POTSI WRVAS SRS-22	Core stabilization exercises and brace	Scientific exercises approach to scoliosis and brace
Lin et al., 2020 [34]	Hong-Kong	23 (0/23)	10–14	Risser Sign 0 to 2	Yes	SRS-22r SAQ BrQ	Conventional orthosis	Automated pressure-adjustable orthosis
Trzcińska et al., 2020 [35]	Poland	60 (0/60)	11–15	Risser Sign < 5	Yes	Posture analysis using Zebris CMS10 system	FITS method according to Białek and M’hango.	FED method
Dufvenberg et al., 2021 [36]	Sweden	135 (24 /111)	9–17	NA	Yes	ATR IPAQ-SF SAQ (pictures) SRS-22r EQ-5D-Y Treatment adherence	Physical activity	I1: Boston night brace I2: Scoliosis-specific exercise
Kocaman et al., 2021 [37]	Turkey	28 (7/21)	10–18	Risser Sign ≤ 3	Yes	ATR WRVAS Spinal mobility SRS-22 Muscle strength	Core stabilization exercises	Schroth exercises
Mohamed et al., 2021 [38]	Egypt	32 (0/32)	14–16	Risser Sign 2–5	Yes	ATR Static plantar pressure distribution 6 MWT	PNF exercises	Schroth exercises
Abdel-Aziem et al., 2022 [39]	Saudi Arabia	52 (15/37)	10–18	NA	Yes	Surface topographyDynamic postural stability	Schroth exercises	Hippotherapy in combination with Schroth exercises
Akyurek et al., 2022 [40]	Turkey	29 (0/29)	10–17	Risser Sign ≤ 3	Yes	ATR Spine joint position sense Posturescreen mobile POTSI ATSI WRVAS	Waiting list	Schroth exercises
Lin et al., 2022 [41]	China	30 (0/30)	10–14	Risser Sign 0–2	Yes	SRS-22r TAPS BrQ	Conventional orthosis	3D-printed orthosis
Wang et al., 2022 [42]	China	31 (6/25)	10–18	Risser Sign ≤ 3	Yes	ATR Coronal balance index Sagittal balance index SRS-22r	Exercise and brace	Insoles in addition to exercise and brace
Shen et al., 2023 [43]	China	59 (21/38)	10–18	Risser Sign < 5	Yes	ATR SRS-22 Balance	Schroth exercises	Schroth and balance exercises
Zapata et al., 2023 [44]	USA	57 (16/41)	10–16	Risser Sign 0	Yes	Adherence to treatment ATR	Observation	Schroth-based Barcelona Scoliosis Physical Therapy School exercises
Manzak Dursun et al., 2024 [45]	Turkey	32 (1/31)	10–18	Risser Sign 0–5	Yes	ATR Pulmonary functions Respiratory muscle strength Exercise capacity	Pilates-based home exercises	Supervised pilates-based exercises with hybrid telerehabilitation

AVR: apical vertebral rotation, ATR: angle of trunk rotation, Posterior Trunk Symmetry Index (POTSI), cosmetic trunk deformity with the Trunk Appearance Perception Scale (TAPS), and quality of life with the Scoliosis Research Society-22 questionnaire (SRS-22), SAQ: Scoliosis Appearance Questionnaire, IPAQ-SF: International Physical Activity Questionnaire short form, 6 MWT: 6 Minutes Walking Test, WRVAS: The Walter Reed Visual Assessment Scale, PedsQL: Pediatric Quality of Life Inventory, BrQ: The Brace Questionnaire, SRS-23: Scoliosis Research Society-23, EQ-5D-Y: EuroQol 5-Dimensions Youth, CAD/CAM: Computer-Aided Design/ Computer-Aided Manufacturing, TLSO: Thoracolumbosacral Orthosis, PNF: Proprioceptive Neuromuscular Facilitation, I1: Intervention Group 1, I2: Intervention Group 2.

**Table 3:** Methodological quality (PEDro scale score).

	1 *	2	3	4	5	6	7	8	9	10	11	Total
Wong et al., 2005 [24]	Yes	No	No	No	No	No	No	No	Yes	Yes	Yes	3/10
Labelle et al., 2007 [25]	Yes	Yes	Yes	Yes	No	No	No	No	No	Yes	No	4/10
Hasler et al., 2010 [26]	Yes	Yes	Yes	Yes	No	No	Yes	No	No	Yes	Yes	6/10
Weinstein et al., 2013 [6]	No	Yes	No	Yes	No	No	No	No	Yes	Yes	No	4/10
Monticone et al., 2014 [27]	Yes	Yes	Yes	Yes	No	No	Yes	Yes	No	Yes	Yes	7/10
Cobetto et al., 2016 [28]	Yes	Yes	Yes	No	No	Yes	Yes	Yes	No	Yes	Yes	7/10
Kuru et al., 2016 [29]	Yes	Yes	Yes	Yes	No	No	No	Yes	No	Yes	Yes	6/10
Gur et al., 2017 [30]	Yes	Yes	No	Yes	Yes	No	No	Yes	Yes	Yes	Yes	7/10
Yagci et al., 2018 [31]	No	Yes	No	Yes	No	No	Yes	Yes	Yes	Yes	Yes	7/10
Sarkisova et al., 2019 [32]	Yes	Yes	No	Yes	No	No	No	No	No	Yes	No	3/10
Yagci et al., 2019 [33]	Yes	Yes	No	Yes	No	No	Yes	Yes	Yes	Yes	Yes	7/10
Lin et al., 2020 [34]	Yes	No	Yes	No	No	No	No	Yes	No	Yes	Yes	4/10
Trzcińska et al., 2020 [35]	No	Yes	No	Yes	No	No	No	Yes	No	Yes	Yes	5/10
Dufvenberg et al., 2021 [36]	No	Yes	No	Yes	No	No	Yes	Yes	Yes	Yes	Yes	7/10
Kocaman et al., 2021 [37]	Yes	Yes	Yes	Yes	No	No	Yes	Yes	Yes	Yes	Yes	8/10
Mohamed et al., 2021 [38]	No	Yes	Yes	Yes	No	No	Yes	Yes	Yes	Yes	Yes	8/10
Abdel-Aziem et al., 2022 [39]	No	Yes	Yes	Yes	No	No	Yes	Yes	No	Yes	Yes	7/10
Akyurek et al., 2022 [40]	Yes	Yes	No	Yes	No	No	No	Yes	No	Yes	Yes	5/10
Lin et al., 2022 [41]	Yes	Yes	No	Yes	No	No	No	Yes	No	Yes	Yes	5/10
Wang et al., 2022 [42]	Yes	Yes	Yes	Yes	No	No	Yes	Yes	Yes	Yes	Yes	8/10
Shen et al., 2023 [43]	Yes	Yes	No	Yes	No	No	No	Yes	Yes	Yes	Yes	6/10
Zapata et al., 2023 [44]	No	Yes	No	Yes	No	No	Yes	No	No	Yes	Yes	5/10
Manzak Dursun et al., 2024 [45]	Yes	Yes	No	Yes	No	No	Yes	Yes	No	Yes	Yes	6/10

* Note: the ligibility criteria item does not contribute to the total score.

**Table 4:** Physiotherapy Evidence Database (PEDro) scale.

1 *	Eligibility criteria were specified.
2	Subjects were randomly allocated to groups.
3	Allocation was concealed.
4	The groups were similar at baseline regarding the most important prognostic indicators.
5	There was blinding of all subjects.
6	There was blinding of all therapists who administered the therapy.
7	There was blinding of all assessors who measured at least one key outcome.
8	Measures of at least one key outcome were obtained from more than 85% of the subjects initially allocated to groups.
9	All subjects for whom outcome measures were available received the treatment or control condition as allocated, or where this was not the case, data for at least one key outcome were analyzed by “intention to treat”.
10	The results of between-group statistical comparisons are reported for at least one key outcome.
11	The study provides both point measures and measures of variability for at least one key outcome.

* Note: the ligibility criteria item does not contribute to the total score.

## Data Availability

The data presented in this study are available on request from the corresponding author.
